# Genome-wide identification of *PbrbHLH* family genes, and expression analysis in response to drought and cold stresses in pear (*Pyrus bretschneideri*)

**DOI:** 10.1186/s12870-021-02862-5

**Published:** 2021-02-09

**Authors:** Huizhen Dong, Qiming Chen, Yuqin Dai, Wenjie Hu, Shaoling Zhang, Xiaosan Huang

**Affiliations:** State Key Laboratory of Crop Genetics and Germplasm Enhancement, Centre of Pear Engineering Technology Research, Nanjing Agricultual University, Nanjing, China

**Keywords:** Chinese white pear, bHLH TF, Gene family, Evolution, VIGS, Drought stress tolerance, Cold stress tolerance

## Abstract

**Background:**

The basic helix-loop-helix (bHLH) transcription factors play important roles in many processes in plant growth, metabolism and responses to abiotic stresses. Although, the sequence of Chinese white pear genome (cv. ‘Dangshansuli’) has already been reported, there is still a lack of clarity regarding the bHLH family genes and their evolutionary history.

**Results:**

In this work, a genome-wide identification of the *bHLH* genes in Chinese white pear was performed, and we characterized the functional roles of these *PbrbHLH* genes in response to abiotic stresses. Based on the phylogenetic analysis and structural characteristics, 197 identified *bHLH* genes could be well classified into 21 groups. Expansion of *PbrbHLH* gene family was mainly driven by WGD and dispersed duplication with the purifying selection from the recent WGD. The functional annotation enrichment showed that the majority of *PbrbHLHs* were enriched in the GO terms and KEGG pathways involved in responds to stress conditions as TFs. Transcriptomic profiles and qRT-PCR revealed that *PbrbHLH7*, *PbrbHLH8*, *PbrbHLH128*, *PbrbHLH160*, *PbrbHLH161* and *PbrbHLH195* were significantly up-regulated under cold and drought treatments. In addition, *PbrbHLH195*-silenced pear seedlings display significant reduced cold tolerance, exhibiting reduced chlorophyll content, as well as increased electrolyte leakage and concentrations of malondialdehyde and H_2_O_2_.

**Conclusion:**

For the first time, a comprehensive analysis identified the *bHLH* genes in Chinese white pear and demonstrated that *PbrbHLH195* is involved in the production of ROS in response to cold stress, suggesting that members of the *PbrbHLH* family play an essential role in the stress tolerance of pear.

**Supplementary Information:**

The online version contains supplementary material available at 10.1186/s12870-021-02862-5.

## Background

Transcription factors (TFs) are protein molecules with special structure and function of regulating gene expression, which plays many crucial roles in plant growth and development [1]. The basic helix-loop-helix (bHLH) transcription factor family is the second largest family in plants. The members of this family are designated by a highly conserved domain called the bHLH which are able to bind and form DNA dimers [2]. The conserved bHLH domain consists of about 60 amino acids and has two functional segments, the basic region and the HLH region. The N-terminal basic region, which contains 13–17 major basic amino acids, serves as the DNA binding domain to identify and specifically bind to DNA motifs in the promoter of the target gene [3–6]. The HLH region is located at the C-terminus of the bHLH domain, which consists of two parental α-helixes, mainly composed of hydrophobic residues, connected by a relatively dispersed (length and primary sequence) loop region. The HLH domain promotes protein-protein interactions and allows the formation of homo-dimer or hetero-dimer complexes [7]. *bHLH* transcription factors are involved in many process about plant growth and metabolism, such as stomata development [8], light signal transduction [9, 10], flowering regulation [11], anthocyanin and secondary metabolism [12–14]. There have been reported that *bHLH* genes are mainly involved in abiotic stress in plants, such as the responses to drought, low temperature, salt, ABA and mechanical damage [15, 16]. For example, *AtbHLH006, AtbHLH17, AtbHLH32, AtbHLH92, AtbHLH122, AtbHLH128* and *AtbHLH130* are directly or indirectly involved in ABA signaling pathway to improve drought resistance in *Arabidopsis* [17]. The over-expression of *bHLH* TF MYC-type *ICE1*, *ICE2* and *CBF* enhanced the tolerance of *Arabidopsis* to low temperature stress [18]. In wheat, *TabHLH1* is able to regulate ABA-mediated stress tolerance pathway to improve plant adaptability to drought and salt stresses [19]. The *TabHLH39* gene is involved in regulating gene expression levels in stress responses, thereby increasing salt tolerance in over-expressing plants [20]. In rice, *OsbHLH148* and *OsbHLH006* (*RERJ1*) respond to drought stress through the jasmonic acid signaling pathway [21, 22]. The bHLH transcription factor *RsICE1* can improve the cold tolerance of transgenic rice [23]. The expression of the *PebHLH35* gene in populus increased during drought and ABA induction, and *PebHLH35* had an active regulatory effect under drought stress, which mentioned plant tolerance [24]. Similarly, it was shown that VabHLH1 and VvbHLH1 are positive regulators of response to low temperature stress in Chinese wild *Vitis amurensis* and *Vitis vinifera* cv. Cabernet Sauvignon, and able to confer enhanced low temperature tolerance to transgenic plants by regulating the expression level of cold regulated (*COR*) genes [25].

To date, based on the rapid development of genome sequencing, a number of plant bHLH TF genes have been identified and characterized in many species. Although, there are 162, 167, 155, 124 and 188 *bHLHs* have been identified in *Arabidopsis,* rice, bean, potato and apple, respectively [26], there has been no report about the *bHLH* family in pear. Pear is an important cash crop and widely distributed in the world. However, pears were suffered from abiotic stresses such as drought, low temperature, and salt during the growth and development process, which not only restricts the cultivation area, but also affects their growth, development and yield. Therefore, investigating of pear bHLH transcription factors are necessary to elucidate the biological processes underlying pear stress responses.

In this study, we identified 197 pear *bHLH* (*PbrbHLH*) genes from the Chinese white pear genomic sequence and carried out phylogenetic analysis to determine the relationships among these genes. Analysis results of protein motifs and intron/exon structures support the classification of the *bHLH* family. At the same time, we identified duplication events that likely contributed to the expansion of the *bHLH* family. In addition, RNA-Seq data showed that the expression patterns of *PbrbHLHs* differed in response to drought and cold stresses. The data from this study will increase our understanding of *PbrbHLH* functions associated with stress responses. Meanwhile, our systematic analysis provided a foundation for further mechanisms of cold-tolerance and drought-tolerance for *bHLH* genes in pear, especially for aiming to identify candidate genes that may be involved in the cold- and drought-tolerance of pears.

## Results

### Identification, classification and function annotation of *bHLH* genes in Chinese white pear

To identify the *PbrbHLH* genes, we performed local HMM-search with the HMM file (PF00010) against Chinese white pear genome, with default parameters. 200 putative PbrbHLH protein sequences were identified. SMART and NCBI Batch CD-Search tools were used to confirm the existence of the conserved bHLH domain, and redundant sequences were removed. We finally obtained 197 sequences in pear *bHLH* family. According to the order of gene ID, these genes were named from *PbrbHLH1* to *PbrbHLH197* (Table 1 and Table S1). 168 *PbrbHLH* genes are randomly distributed on all 17 chromosomes ranging from 1 to 25 per chromosome, and the others were localized to 25 unanchored scaffolds (Table 1). Chromosome 15 has the most *PbrbHLHs* (25 genes), followed by chr 5 (21 genes) and chr 14 (15 genes).

**Table 1:** Characteristics of identified PbrbHLH proteins

Gene name	Best hit in AT	Chr	start	end	ORF length	Extron num	MW (kDa)	PI	GRAVY
*PbrbHLH1*	*AT5G54680.1*	Chr5	27,094,335	27,096,404	720	5	26.13	6.29	−0.575
*PbrbHLH2*	*AT4G21330.1*	Chr5	27,016,336	27,016,952	441	3	16.28	4.24	−0.254
*PbrbHLH3*	*AT1G61660.3*	Chr5	26,993,507	27,001,224	1323	5	47.48	4.74	−0.502
*PbrbHLH4*	*AT2G40200.1*	Chr5	25,158,927	25,161,863	804	2	28.85	9.06	−0.296
*PbrbHLH5*	*AT1G51140.1*	Chr3	18,537,202	18,539,580	1254	6	45.25	6.24	−0.758
*PbrbHLH6*	*AT1G35460.1*	Chr15	40,505,769	40,508,649	657	4	23.32	5.67	−0.565
*PbrbHLH7*	*AT5G57150.3*	scaffold1040.0	66,902	68,814	774	5	29.31	8.72	−0.577
*PbrbHLH8*	*AT5G65640.1*	Chr8	6,468,092	6,469,857	1089	4	40.74	4.32	−0.569
*PbrbHLH9*	*AT5G01305.1*	Chr12	16,099,618	16,100,214	600	1	22.23	6.7	−0.57
*PbrbHLH10*	*AT1G06690.1*	Chr17	15,208,210	15,222,427	2412	15	88.59	9.32	−0.575
*PbrbHLH11*	*AT4G29100.1*	Chr17	16,366,147	16,368,813	1026	9	37.25	7.55	−0.702
*PbrbHLH12*	*AT2G14760.1*	scaffold1151.0	9562	11,543	1062	5	38.46	5.25	−0.539
*PbrbHLH13*	*AT4G33880.1*	scaffold1151.0	36,152	38,137	1104	5	40.15	4.94	− 0.688
*PbrbHLH14*	*AT1G35460.1*	Chr8	5,724,025	5,727,879	702	4	24.66	8.63	−0.676
*PbrbHLH15*	*AT4G02590.2*	Chr12	2,040,321	2,045,714	1014	7	35.47	6.24	−0.44
*PbrbHLH16*	*AT5G43650.1*	scaffold1203.0	77,475	79,190	546	3	21.27	10.1	−0.846
*PbrbHLH17*	*AT5G65640.1*	Chr15	20,949,598	20,951,150	1077	4	39.71	4.95	−0.481
*PbrbHLH18*	*AT5G41315.1*	scaffold1226.0	3	1454	690	3	25.9	5.7	−0.672
*PbrbHLH19*	*AT5G65640.1*	Chr15	35,299,620	35,301,589	1098	4	41.14	4.39	−0.585
*PbrbHLH20*	*AT2G42280.3*	Chr2	9,337,765	9,339,806	1278	6	47.26	6.38	−0.893
*PbrbHLH21*	*AT5G41315.3*	Chr14	10,423,756	10,458,851	2358	9	87.65	5.13	−0.457
*PbrbHLH22*	*AT5G41315.3*	Chr14	10,657,342	10,660,577	2052	8	76.58	5.91	−0.422
*PbrbHLH23*	*AT1G27660.1*	Chr15	3,038,629	3,044,314	1371	7	49.35	6.99	−0.467
*PbrbHLH24*	*AT1G09530.5*	Chr16	10,077,360	10,081,240	2154	7	76.57	6.55	−0.614
*PbrbHLH25*	*AT3G06120.1*	scaffold132.0.1	335,925	337,641	621	3	22.95	9.23	−0.194
*PbrbHLH26*	*AT1G72210.1*	scaffold132.0.1	415,469	417,397	981	3	36.18	6.51	−0.448
*PbrbHLH27*	*AT1G72210.1*	Chr4	632,585	633,725	783	3	29.21	5.83	−0.361
*PbrbHLH28*	*AT1G09530.5*	Chr4	2,380,472	2,383,676	2145	7	76.42	6.51	− 0.679
*PbrbHLH29*	*AT5G08130.5*	Chr14	14,953,088	14,955,645	1713	9	62.47	9.04	−0.704
*PbrbHLH30*	*AT4G00050.1*	Chr14	15,502,751	15,505,135	897	6	32.66	9.6	−0.811
*PbrbHLH31*	*AT2G24260.2*	Chr2	20,591,591	20,595,907	1410	7	48.56	6.66	−0.474
*PbrbHLH32*	*AT3G22100.1*	Chr17	16,734,318	16,735,664	1338	1	48.69	9.24	−0.584
*PbrbHLH33*	*AT2G46810.3*	Chr1	1,364,775	1,367,503	1302	4	48.52	6.03	−0.662
*PbrbHLH34*	*AT2G41130.1*	scaffold1497.0	3118	4544	726	2	27.16	8.62	−0.54
*PbrbHLH35*	*AT2G41130.1*	scaffold1497.0	33,505	34,931	726	2	27.16	8.62	−0.54
*PbrbHLH36*	*AT1G49770.1*	Chr5	9,948,267	9,951,160	849	3	30.42	9.21	−0.403
*PbrbHLH37*	*AT3G21330.1*	Chr9	9,245,860	9,247,209	1353	1	50.11	6.82	−0.71
*PbrbHLH38*	*AT4G16430.1*	Chr10	9,856,487	9,857,979	1425	2	52.65	7.24	−0.482
*PbrbHLH39*	*AT4G16430.1*	Chr10	9,876,054	9,878,083	1512	1	55.69	6.16	−0.454
*PbrbHLH40*	*AT4G02590.2*	Chr10	10,112,702	10,116,115	894	6	31.43	6.41	−0.368
*PbrbHLH41*	*AT5G41315.3*	Chr6	7,815,415	7,818,898	1878	6	70.05	6.01	−0.466
*PbrbHLH42*	*AT2G40200.1*	Chr1	7,131,102	7,132,823	831	2	29.84	9.37	−0.358
*PbrbHLH43*	*AT2G43650.1*	scaffold162.0	146,901	178,353	2154	19	80.5	4.75	−0.807
*PbrbHLH44*	*AT3G26744.4*	Chr14	12,125,142	12,127,506	1605	4	57.59	5.52	−0.497
*PbrbHLH45*	*AT5G50915.1*	Chr15	13,329,386	13,332,068	1068	7	39.35	7.02	−0.775
*PbrbHLH46*	*AT3G26744.4*	Chr15	13,269,686	13,272,442	1644	4	59.07	5.68	−0.52
*PbrbHLH47*	*AT5G53210.1*	Chr13	217,187	219,497	1260	3	44.87	5.92	−0.355
*PbrbHLH48*	*AT2G40200.1*	Chr7	11,517,886	11,519,641	858	2	31.01	8.33	−0.285
*PbrbHLH49*	*AT2G31280.1*	Chr10	8,959,774	8,964,350	2223	11	81.76	6.18	−0.364
*PbrbHLH50*	*AT3G47640.1*	Chr6	1,652,351	1,654,266	744	4	27.31	7.55	−0.745
*PbrbHLH51*	*AT3G28857.1*	Chr6	1,630,526	1,630,925	330	2	12.16	6.96	−0.443
*PbrbHLH52*	*AT3G47710.1*	Chr6	1,573,126	1,574,101	282	2	10.38	9.4	−0.685
*PbrbHLH53*	*AT5G62610.2*	Chr6	1,235,430	1,238,083	870	6	30.55	5.08	−0.712
*PbrbHLH54*	*AT3G14270.1*	Chr2	17,666,963	17,682,155	7410	18	274.01	5.39	−0.488
*PbrbHLH55*	*AT3G19500.1*	Chr10	15,027,760	15,030,379	783	5	27.99	8.59	−0.544
*PbrbHLH56*	*AT1G69010.1*	Chr3	21,842,280	21,845,477	1008	7	36.78	5.88	−0.869
*PbrbHLH57*	*AT1G68920.3*	Chr3	21,774,132	21,778,787	2283	11	83	7.4	−0.5
*PbrbHLH58*	*AT1G68810.1*	Chr3	21,442,039	21,445,012	1059	2	39.09	6.76	−0.628
*PbrbHLH59*	*AT1G25330.1*	Chr3	20,976,835	20,978,516	747	6	27.71	7.42	−0.593
*PbrbHLH60*	*AT2G20180.7*	Chr3	20,788,185	20,790,941	1113	6	40.55	8.21	−0.433
*PbrbHLH61*	*AT5G53210.1*	Chr1	9,416,218	9,418,685	1203	3	43.2	4.99	−0.386
*PbrbHLH62*	*AT4G17880.1*	Chr16	9,798,223	9,800,129	1563	2	58.32	6.68	−0.529
*PbrbHLH63*	*AT3G07340.1*	scaffold202.0.1	171,486	173,848	1632	7	58.63	7.43	−0.613
*PbrbHLH64*	*AT1G22490.1*	Chr6	19,415,337	19,417,849	969	3	35.91	9.29	−0.526
*PbrbHLH65*	*AT2G41130.1*	Chr17	10,057,378	10,058,791	726	2	27.1	8.01	−0.536
*PbrbHLH66*	*AT1G66470.1*	Chr9	6,731,103	6,744,452	1836	9	66.09	8.63	−0.445
*PbrbHLH67*	*AT3G07340.1*	Chr10	12,766,002	12,768,825	1713	8	61.84	7.14	−0.755
*PbrbHLH68*	*AT4G29100.1*	Chr15	15,967,308	15,972,064	1176	9	42.2	7.3	−0.576
*PbrbHLH69*	*AT5G56960.2*	Chr15	14,814,368	14,817,543	1761	7	65.78	8.39	−0.418
*PbrbHLH70*	*AT1G68810.1*	Chr10	3,695,569	3,699,378	810	2	29.05	8.08	−0.275
*PbrbHLH71*	*AT3G07340.1*	Chr4	11,760,522	11,763,609	1587	8	56.76	8.19	−0.6
*PbrbHLH72*	*AT1G69010.1*	Chr17	17,757,708	17,762,671	1221	7	43.88	8.35	− 0.53
*PbrbHLH73*	*AT2G31210.1*	Chr2	10,054,241	10,056,179	1542	3	56.71	5.83	−0.586
*PbrbHLH74*	*AT2G31210.1*	Chr2	10,074,575	10,076,304	1395	3	51.69	6.47	−0.646
*PbrbHLH75*	*AT2G31210.1*	Chr2	10,457,137	10,458,866	1395	3	51.69	6.47	−0.646
*PbrbHLH76*	*AT2G31210.1*	Chr2	10,477,477	10,479,415	1542	3	56.71	5.83	−0.586
*PbrbHLH77*	*AT1G72210.1*	Chr12	721,524	723,015	975	3	36.53	5	−0.442
*PbrbHLH78*	*AT4G09820.1*	Chr15	25,886,991	25,893,792	2013	7	74.31	4.9	−0.561
*PbrbHLH79*	*AT3G07340.2*	Chr13	6,771,090	6,772,369	543	6	19.93	8.49	−0.388
*PbrbHLH80*	*AT4G17880.1*	Chr1	4,498,172	4,499,991	1494	1	55.23	5.61	−0.508
*PbrbHLH81*	*AT5G53210.1*	Chr1	9,180,049	9,182,549	1203	3	43.18	4.99	−0.393
*PbrbHLH82*	*AT5G01305.1*	Chr4	3,361,294	3,361,890	600	1	22.23	6.7	−0.57
*PbrbHLH83*	*AT3G50330.1*	Chr15	7,854,064	7,854,920	756	1	27.88	9.35	−0.562
*PbrbHLH84*	*AT1G49770.1*	Chr15	7,767,619	7,768,847	705	3	25.85	6.73	−0.308
*PbrbHLH85*	*AT4G36930.1*	Chr15	7,762,918	7,765,404	1056	6	38.16	5.96	−0.572
*PbrbHLH86*	*AT2G14760.1*	Chr15	7,261,485	7,263,160	1077	4	39.97	5.3	−0.758
*PbrbHLH87*	*AT1G10120.2*	Chr15	6,913,207	6,915,155	1281	7	45.8	6.29	−0.661
*PbrbHLH88*	*AT5G08130.7*	Chr6	4,286,812	4,291,392	1773	11	65.08	8.94	−0.788
*PbrbHLH89*	*AT1G73830.1*	Chr6	3,710,462	3,711,993	795	6	29.47	5.21	−0.644
*PbrbHLH90*	*AT3G20640.1*	Chr15	9,143,054	9,146,117	1368	7	49.92	6.44	−0.702
*PbrbHLH91*	*AT2G34820.1*	Chr10	2,735,718	2,736,795	930	2	35.01	4.61	−0.492
*PbrbHLH92*	*AT3G26744.4*	Chr17	23,941,072	23,943,930	1536	4	55.71	4.83	−0.496
*PbrbHLH93*	*AT2G14760.1*	Chr8	12,990,009	12,991,896	1086	4	40.29	4.99	−0.785
*PbrbHLH94*	*AT1G69010.1*	Chr9	18,756,829	18,761,640	1134	7	40.78	6.44	−0.647
*PbrbHLH95*	*AT4G34530.1*	Chr10	21,887,740	21,890,367	1320	7	48.14	5.75	−0.584
*PbrbHLH96*	*AT4G34530.1*	Chr10	21,977,209	21,979,827	1320	7	48.19	5.75	−0.599
*PbrbHLH97*	*AT4G37850.1*	Chr5	7,214,750	7,216,451	1029	4	38.08	7.61	−0.353
*PbrbHLH98*	*AT4G37850.1*	Chr5	7,248,643	7,250,344	1029	4	38.08	7.61	−0.353
*PbrbHLH99*	*AT4G14410.2*	scaffold351.0.1	189,513	191,335	675	4	24.94	5.45	−0.665
*PbrbHLH100*	*AT1G51140.1*	Chr11	23,652,606	23,656,188	1305	6	47.29	6.74	−0.775
*PbrbHLH101*	*AT3G19860.1*	Chr2	16,242,907	16,244,464	975	5	35.96	7.99	−0.951
*PbrbHLH102*	*AT4G17880.1*	Chr11	28,888,209	28,890,472	1386	2	50.93	6.99	−0.374
*PbrbHLH103*	*AT2G42280.1*	Chr7	453,441	455,564	1308	6	48.48	7.48	−0.905
*PbrbHLH104*	*AT5G50915.1*	Chr12	13,203,431	13,205,370	1071	7	39.45	8.36	−0.654
*PbrbHLH105*	*AT4G37850.1*	Chr15	25,516,823	25,518,252	1062	3	39.06	6.42	−0.49
*PbrbHLH106*	*AT4G37850.1*	Chr15	25,435,077	25,436,506	1062	3	39.06	6.42	−0.49
*PbrbHLH107*	*AT5G37800.1*	Chr17	7,619,610	7,621,431	792	6	29.3	7.97	−0.852
*PbrbHLH108*	*AT4G34530.1*	Chr5	7,750,598	7,753,931	1272	7	46.43	6.01	−0.602
*PbrbHLH109*	*AT5G67060.2*	Chr2	13,406,418	13,407,251	837	1	31.15	10.34	−0.595
*PbrbHLH110*	*AT4G36930.1*	Chr2	13,319,368	13,322,043	1122	8	40.81	6.09	−0.537
*PbrbHLH111*	*AT4G02590.2*	Chr5	19,146,494	19,150,070	909	6	31.91	6.05	−0.313
*PbrbHLH112*	*AT4G16430.1*	Chr5	18,934,846	18,936,967	1512	1	55.88	6.37	−0.442
*PbrbHLH113*	*AT4G16430.1*	Chr5	18,869,375	18,870,585	1128	2	41.25	7	−0.454
*PbrbHLH114*	*AT2G31280.1*	Chr5	18,633,552	18,637,955	2229	11	82.45	6.31	−0.373
*PbrbHLH115*	*AT1G09530.5*	Chr12	3,917,496	3,921,379	2154	7	76.57	6.55	−0.614
*PbrbHLH116*	*AT2G28160.2*	Chr3	14,840,221	14,842,265	1053	4	38.35	6.73	−0.396
*PbrbHLH117*	*AT1G69550.1*	Chr3	14,857,124	14,874,269	3528	9	134.38	7.71	−0.307
*PbrbHLH118*	*AT2G28160.2*	Chr3	14,886,366	14,888,189	1044	4	38.07	6.05	−0.405
*PbrbHLH119*	*AT4G20970.1*	Chr17	3,759,373	3,760,370	666	3	25.05	7.66	−0.583
*PbrbHLH120*	*AT4G20970.1*	Chr17	3,775,165	3,776,020	627	3	24.01	9.09	−0.656
*PbrbHLH121*	*AT3G26744.4*	Chr14	12,762,203	12,765,393	1605	4	57.59	5.52	−0.497
*PbrbHLH122*	*AT5G50915.1*	Chr14	12,855,296	12,858,003	1071	7	39.5	8.57	−0.669
*PbrbHLH123*	*AT3G20640.1*	Chr5	13,842,159	13,845,048	1371	7	49.94	6.36	−0.701
*PbrbHLH124*	*AT5G54680.1*	Chr5	13,525,331	13,527,890	693	5	25.14	9.13	−0.554
*PbrbHLH125*	*AT2G16910.1*	Chr5	13,127,432	13,130,656	1695	8	64.04	5.43	−0.692
*PbrbHLH126*	*AT2G16910.1*	Chr5	13,122,299	13,123,691	786	6	29.37	7.86	−0.181
*PbrbHLH127*	*AT2G16910.1*	Chr5	13,117,288	13,120,346	1818	8	67.88	4.9	−0.707
*PbrbHLH128*	*AT1G01260.2*	Chr1	3,882,499	3,884,328	1833	1	67.36	6.64	−0.512
*PbrbHLH129*	*AT1G68810.1*	Chr13	2,460,589	2,463,318	1053	2	38.75	6.83	−0.656
*PbrbHLH130*	*AT4G29100.1*	Chr2	19,901,470	19,905,690	1140	9	40.95	6.9	−0.604
*PbrbHLH131*	*AT2G31730.2*	scaffold467.0	196,436	199,525	870	9	32.43	7.61	−0.638
*PbrbHLH132*	*AT5G01305.1*	Chr12	5,216,197	5,216,784	591	1	21.8	8.46	−0.663
*PbrbHLH133*	*AT3G21330.1*	Chr17	9,031,563	9,032,906	1347	1	49.3	6.9	−0.605
*PbrbHLH134*	*AT1G25330.1*	Chr13	1,982,008	1,983,683	717	6	26.63	5.98	−0.684
*PbrbHLH135*	*AT2G43010.2*	Chr9	13,138,658	13,140,883	1674	7	61.03	6.95	−0.725
*PbrbHLH136*	*AT1G69010.1*	Chr13	4,199,817	4,203,034	1005	7	36.54	6.59	−0.843
*PbrbHLH137*	*AT1G68920.3*	Chr13	4,107,727	4,111,792	1653	8	58.78	5.06	−0.582
*PbrbHLH138*	*AT4G00050.1*	Chr6	4,887,155	4,889,553	1131	5	41.25	7.74	−0.666
*PbrbHLH139*	*AT3G56970.1*	scaffold526.0	20,152	21,309	759	3	28.92	8.42	−0.695
*PbrbHLH140*	*AT3G24140.2*	Chr4	12,461,187	12,462,781	1365	3	50.78	4.9	−0.598
*PbrbHLH141*	*AT1G05805.1*	Chr4	11,676,123	11,681,214	1086	6	38.44	8.86	−0.638
*PbrbHLH142*	*AT5G53210.1*	Chr11	12,922,232	12,924,549	1260	3	44.84	5.43	−0.354
*PbrbHLH143*	*AT2G31730.2*	Chr7	10,672,016	10,674,734	861	9	32.14	9.07	−0.581
*PbrbHLH144*	*AT2G20180.6*	Chr5	17,244,166	17,248,840	1464	9	53.02	9.9	−0.765
*PbrbHLH145*	*AT3G06120.1*	scaffold639.0.1	60,715	62,445	621	3	22.95	9.23	−0.194
*PbrbHLH146*	*AT2G24260.1*	Chr15	11,765,124	11,768,866	1413	7	48.48	6.56	−0.485
*PbrbHLH147*	*AT5G50010.1*	scaffold655.0	182,209	184,247	1083	1	39.75	4.43	−0.736
*PbrbHLH148*	*AT3G19860.1*	Chr13	14,656,478	14,658,132	1059	5	38.54	7.33	−0.966
*PbrbHLH149*	*AT2G27230.2*	Chr6	10,899,693	10,903,475	2310	11	84.66	4.58	−0.365
*PbrbHLH150*	*AT4G00870.1*	Chr6	11,224,408	11,226,263	1542	2	57.19	7.2	−0.605
*PbrbHLH151*	*AT5G67060.1*	Chr15	23,381,295	23,382,023	732	1	26.85	10.62	−0.605
*PbrbHLH152*	*AT4G36930.1*	scaffold697.0	138,071	140,687	1167	8	42.28	5.39	−0.484
*PbrbHLH153*	*AT3G53690.1*	Chr2	16,659,400	16,666,799	2487	9	92.52	4.9	−0.496
*PbrbHLH154*	*AT5G61270.1*	scaffold745.0.1	145,406	150,762	1320	6	47.07	7.27	−0.482
*PbrbHLH155*	*AT4G17880.1*	Chr14	19,188,604	19,190,693	1449	2	53.93	6.75	−0.515
*PbrbHLH156*	*AT4G17880.1*	Chr14	19,223,454	19,225,368	1566	2	58.53	6.46	−0.578
*PbrbHLH157*	*AT2G27230.2*	Chr14	19,633,839	19,638,140	2385	10	87.47	4.82	−0.343
*PbrbHLH158*	*AT3G53690.1*	Chr15	17,135,114	17,139,758	1623	7	60.85	7.13	−0.471
*PbrbHLH159*	*AT3G21330.1*	scaffold763.0.1	64,350	65,696	1350	1	49.94	6.93	−0.709
*PbrbHLH160*	*AT5G57150.1*	Chr16	15,621,565	15,625,703	735	5	27.77	5	−0.444
*PbrbHLH161*	*AT1G32640.1*	scaffold773.0	23,504	24,874	1374	1	50.16	6.44	−0.657
*PbrbHLH162*	*AT5G65640.1*	scaffold775.0	130,165	131,652	1074	4	39.49	4.51	−0.461
*PbrbHLH163*	*AT1G68810.1*	Chr15	38,735,037	38,735,835	708	2	25.8	9.79	−0.708
*PbrbHLH164*	*AT5G10530.1*	Chr11	4,864,156	4,884,598	2229	4	82.36	5.41	−0.253
*PbrbHLH165*	*AT3G19500.1*	scaffold809.0	153,215	155,872	783	5	27.77	8.3	−0.491
*PbrbHLH166*	*AT4G00870.1*	Chr6	6,218,911	6,220,570	1410	2	52.11	6.03	−0.393
*PbrbHLH167*	*AT5G46830.1*	Chr6	6,266,723	6,268,150	1137	2	41.84	6.79	−0.353
*PbrbHLH168*	*AT2G42280.1*	Chr14	20,106,973	20,109,719	951	6	34.74	9.18	−0.707
*PbrbHLH169*	*AT3G57800.2*	Chr14	20,181,881	20,186,582	1110	8	40.67	5.6	−0.443
*PbrbHLH170*	*AT1G73830.2*	Chr14	16,070,706	16,072,305	798	6	29.51	5.21	−0.549
*PbrbHLH171*	*AT1G59640.1*	Chr13	3,371,476	3,373,746	891	6	32.07	6.88	−0.747
*PbrbHLH172*	*AT1G29950.2*	Chr10	6,704,729	6,706,250	837	2	30.51	5.21	−0.485
*PbrbHLH173*	*AT4G38070.1*	Chr15	26,803,608	26,804,342	636	2	23.13	10.48	−0.321
*PbrbHLH174*	*AT5G67060.2*	Chr15	23,542,843	23,543,574	735	1	26.97	10.62	−0.584
*PbrbHLH175*	*AT4G29100.1*	Chr17	8,804,002	8,806,668	1026	9	37.25	7.55	−0.702
*PbrbHLH176*	*AT2G28160.1*	Chr3	14,083,787	14,085,285	972	4	35.39	4.46	−0.457
*PbrbHLH177*	*AT1G72210.1*	Chr15	37,042,350	37,044,909	936	3	34.87	7.34	−0.613
*PbrbHLH178*	*AT5G50010.1*	Chr15	37,570,541	37,573,248	1083	1	39.82	4.48	−0.755
*PbrbHLH179*	*AT2G31220.1*	Chr7	3,923,753	3,925,356	870	4	32.13	6.95	−0.564
*PbrbHLH180*	*AT2G31210.1*	Chr7	3,929,075	3,931,367	1545	3	56.99	5.97	−0.643
*PbrbHLH181*	*AT4G37850.1*	scaffold911.0	70,566	72,288	1044	4	38.53	7.87	−0.403
*PbrbHLH182*	*AT4G37850.1*	scaffold911.0	148,794	150,516	1044	4	38.53	7.87	−0.403
*PbrbHLH183*	*AT5G43650.1*	scaffold914.0	94,761	97,214	756	3	29.2	8.99	−0.766
*PbrbHLH184*	*AT1G30670.1*	Chr5	20,425,616	20,427,088	924	2	34.42	5.16	−0.385
*PbrbHLH185*	*AT3G19860.1*	Chr5	10,201,972	10,206,005	1029	5	38.34	6.43	−0.934
*PbrbHLH186*	*AT5G62610.2*	scaffold927.0	32,483	35,092	870	6	30.54	5.24	−0.69
*PbrbHLH187*	*AT4G20970.1*	scaffold930.0	119,533	120,307	507	3	18.18	5.28	−0.21
*PbrbHLH188*	*AT5G41315.3*	Chr6	13,870,216	13,873,451	1986	8	73.87	5.34	−0.472
*PbrbHLH189*	*AT3G19500.1*	scaffold939.0	83,047	86,537	804	5	28.76	8.86	−0.513
*PbrbHLH190*	*AT5G56960.2*	Chr2	18,541,013	18,544,243	1791	8	67.08	8.89	−0.375
*PbrbHLH191*	*AT4G37850.1*	Chr16	18,387,503	18,388,918	1056	3	38.73	7.31	−0.474
*PbrbHLH192*	*AT4G37850.1*	Chr16	18,414,150	18,415,660	1062	3	38.85	6.51	−0.484
*PbrbHLH193*	*AT2G14760.1*	Chr10	16,254,387	16,256,363	1062	5	38.62	6.31	−0.526
*PbrbHLH194*	*AT2G34820.1*	Chr5	23,493,559	23,495,765	1134	2	42.55	5.64	−0.085
*PbrbHLH195*	*AT1G32640.1*	scaffold984.0	37,062	39,652	2067	1	75.5	5.8	−0.61
*PbrbHLH196*	*AT2G42280.1*	Chr14	4,887,436	4,890,120	999	5	36.51	9.64	−0.612
*PbrbHLH197*	*AT3G57800.2*	Chr14	4,960,408	4,965,046	1050	6	38.32	6.01	−0.581

**Fig. 1:**
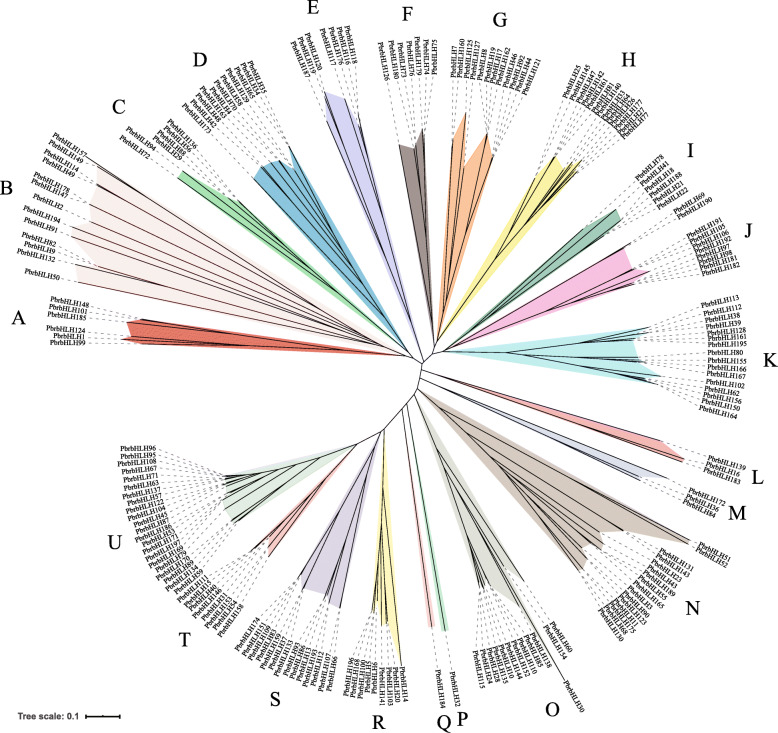
Un-rooted phylogenetic tree of PbrbHLH proteins.MEGA 7 was used to construct the phylogenetic tree based on the protein sequences. iTOL was used to annotate and review the phylogenic tree. The proteins were clustered into 21 groups. Different background colors indicate the different group of the PbrbHLH proteins

**Fig. 2:**
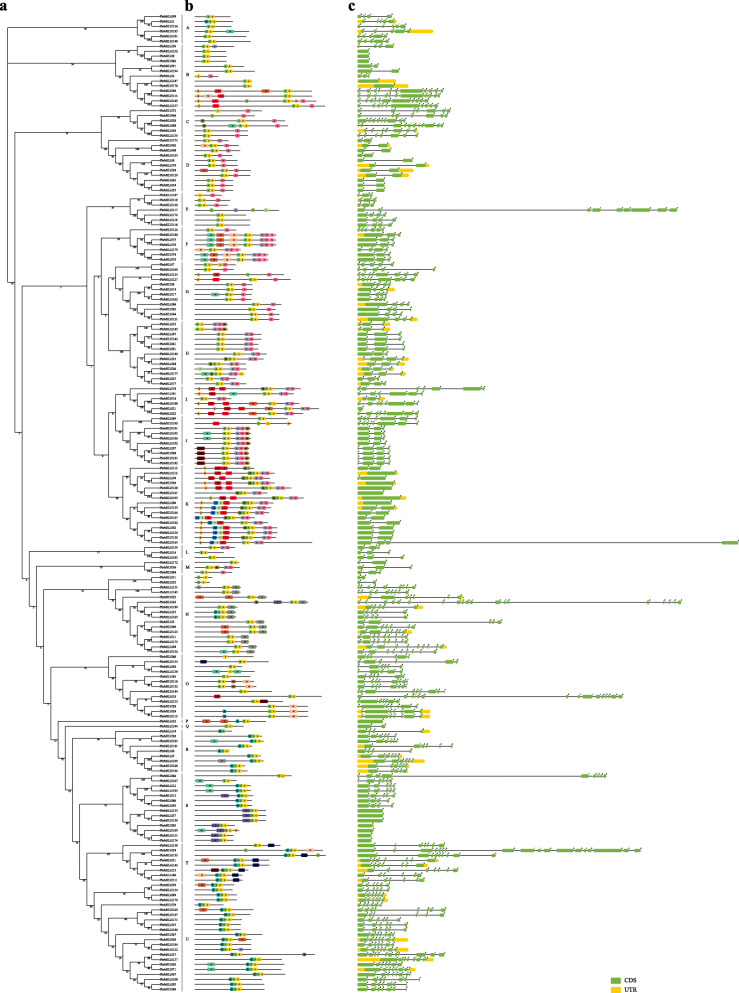
Schematics of the gene structure and conserved motifs in the *PbrbHLH* family. **a** Subgroup classification. Phylogenetic tree was generated using 197 *PbrbHLH* genes with MEGA7. The subgroup names were labeled accordingly. **b** Conserved motif analysis. Twenty distinct motifs were identified with MEME suite and each motif was represented with different color. **c**. Gene structural analysis

The exact number of subgroup classifications for plant bHLH proteins is unknown, but is thought to be 15–32 [7, 8, 27]. To classify these genes and investigate their evolutionary relationships, phylogenetic tree was built applying the NJ method (Fig. 1, Fig. 2a and Fig. S1). The unrooted tree revealed that *PbrbHLH* gene family could be separated into 21 clades with the subfamily names A to U, which is the same number as those found in tomato [28] and *Phaseolus vulgaris* [29]. Unlike other clades, clade P and Q contained a single bHLH protein, respectively, meaning that *PbrbHLH32* and *PbrbHLH184* are unique. Furthermore, the NJ-tree built with these two PbrbHLHs and 167 AtbHLHs protein sequences indicated that the correlation between PbrbHLH132 and PbrbHLH184 and other bHLH proteins were relatively low (Fig. S1), which is consistent with the un-rooted tree. Except clade P and Q, the gene numbers of each clade varied wildly from 3 (clade L and M) to 22 (clade U). The results of gene structure analysis also showed that the *PbrbHLH* gene family have a broad range of exon numbers as well the gene structural diversity (Fig. 2c), such as the fact that there is no characteristic distribution pattern of exons and UTRs within most of certain subfamilies. However, the distribution patterns of exons were relatively conserved in clade D, F, G, H, J, K and U, and genes in these clades have a high similarity in exons number, exon pattern and the length of each exon, such as *PbrbHLH73*, *PbrbHLH74*, *PbrbHLH75*, *PbrbHLH76* and *PbrbHLH180* in clade F and *PbrbHLH47* to *PbrbHLH77* in clade H.

The characteristics of the *PbrbHLH* family and their coding genes are shown in Table 1 and Table S1. The protein molecular weights of bHLHs were from 10.38 to 274.01 kD. Protein isoelectric points (pI) ranged from 4.24 to 10.62, and 120 of them were lower than 7 (Table 1). The grand average of hydropathy (GRAVY) for all bHLH proteins in pear was positive, suggesting that all PbrbHLHs were likely soluble proteins which are consistent with their potential functions as TFs.

The annotation information from GO and KEGG databases were able to depict potential function of these genes. To predict the functions of identified *PbrbHLH* genes, the functional enrichment analysis of PbrbHLHs and a blastp analysis against the protein sequences of reported *AtbHLH* genes were all performed. As shown in Fig. S2a and Table S1, *PbrbHLHs* were mainly enriched in the terms of binding, cell part, cellular process, metabolic process and some regulation function, and all of these functions and processes are closely related to TFs. In addition, the KEGG enrichment result showed that these genes were largely enriched in circadian rhythm, MAPK signaling and plant hormone signal transduction pathways (Fig. S2b), all of which are the main mechanisms by which *bHLH* family TFs regulate the expression of downstream genes. Furthermore, the blastp result also showed that the crucial TF of these pathway were detected, including *AtbHLH004* (the orthologous of*PbrbHLH62*, *PbrbHLH80*, *PbrbHLH102*, *PbrbHLH155*, *PbrbHLH156* and *PbrbHLH162*) and *AtbHLH003* (the orthologous of *PbrbHLH38*, *PbrbHLH39*, *PbrbHLH112* and *PbrbHLH113*), thepositive and negative regulator of jasmonate responses, respectively; *AtbHLH008* (the orthologous of *PbrbHLH24*, *PbrbHLH28*, *PbrbHLH115* and *PbrbHLH135*), a negative regulator of phyB signalling; and *AtbHLH098* (the orthologous of *PbrbHLH47*, *PbrbHLH61*, *PbrbHLH81* and *PbrbHLH142*), a substrate of kinases MPK3 and MPK6.

### Synteny analysis of *PbrbHLHs*

The gene duplication events, such as WGD/segmental duplication, tandem duplication and transposition events, are the main causes for gene family expansion and affect the evolution of protein-coding gene families [30]. By using the MCScanX package, we detected the duplication events of *bHLH* gene family, and each of genes was assigned to one of five different duplication types: singleton, WGD/segmental, tandem, proximal or dispersed. Five duplication types were all detected driving the expansion of the *PbrbHLH* genes (Table 2 and Table S2). The results showed that 58.9% of *bHLH* genes in Chinese white pear were duplicated and retained from WGD/segmental events, and almost one quarter (23.9%) of *PbrbHLHs* was belonged to dispersed type.

**Table 2:** Numbers of bHLH genes from different origins in Chinese white pear

Duplication type	Singleton	Dispersed	Proximal	Tandem	WGD/segmental
No. of *bHLH* genes from different origins (percentage)	3 (1.5)	47 (23.9)	11 (5.6)	20 (10.1)	116 (58.9)

**Fig. 3:**
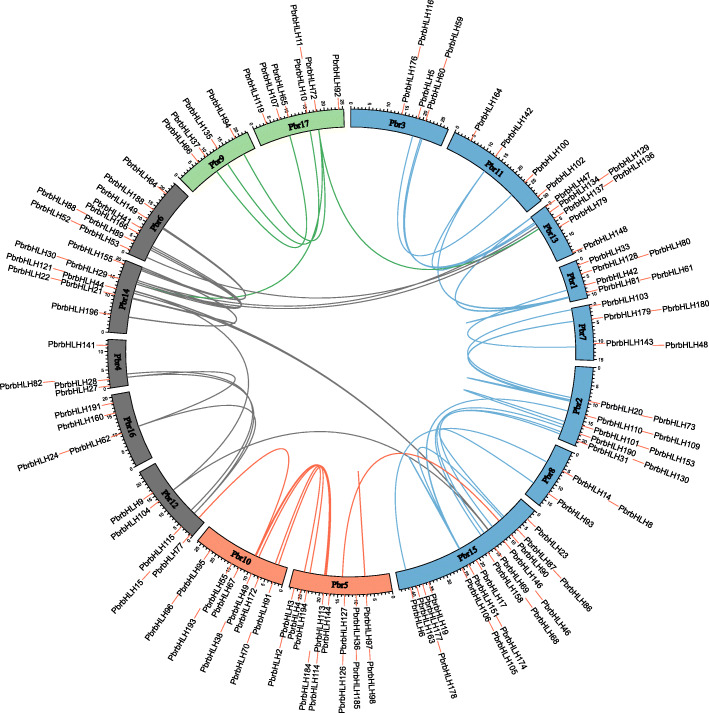
Distribution and collinearity of the *PbrbHLHs*. Red lines along the circumference of the circle mark the positions of genes on chromosomes. The lines in different colors inside the circle indicate collinearity relationships among *PbrbHLH* genes

To explore the evolutionary process behind the *PbrbHLH* genes, we performed intragenomic synteny analysis to identify conservation chromosome blocks within Chinese white pear. The landscape of ortholog *PbrbHLH* genes pairs were shown in Fig. 3 and their chromosomal distribution was random. The evolutionary date of WGD/segmental duplication events could be estimated by the Ks value (synonymous substitutions per site) [31]. As the previous reports, based on Ks values, the genome of pear had undergone two genome-wide duplication events: the ancient WGD (Ks ~ 1.5–1.8) that took place ~ 140 MYA [32] and the recent WGD (Ks ~ 0.15–0.3) occurred at 30–45 MYA [33] in pear. Therefore, we used Ks values to estimate the evolutionary date of the gene duplication events among the *PbrbHLH* gene family. The Ks values implied that most *PbrbHLH* genes were duplicated from a date around the recent WGD event, and some of others were originated from the ancient WGD (Table 3). The selection intensity and direction could be represented by Ka/Ks ratio, Ka/Ks value of one indicates neutral evolution, positive selection was indicated by a Ka/Ks value greater than one, and purifying selection was indicated by a Ka/Ks value less than one [34]. The Ka/Ks ratios of almost all homologous *PbrbHLH* genes were less than one (except the gene pair *PbrbHLH110-PbrbHLH152*), which implying that *PbrbHLHs* mainly evolved under purifying selection (Table 3).

**Table 3:** The duplicate mode and estimation of absolute date for large-scale duplication events for *PbrbHLHs*

Colinearity gene pairs		Duplication type		Method	Ka	Ks	Ka/Ks	MYA	*P*-Value (Fisher)
Gene1	Gene2	Gene1	Gene2						
*PbrbHLH6*	*PbrbHLH14*	WGD	WGD	NG	0.14	0.29	0.47	97.86	0.000549
*PbrbHLH10*	*PbrbHLH135*	WGD	WGD	NG	0.04	0.13	0.30	44.85	6.55E-08
*PbrbHLH20*	*PbrbHLH103*	WGD	WGD	NG	0.08	0.21	0.38	70.19	1.28E-06
*PbrbHLH21*	*PbrbHLH22*	WGD	WGD	NG	NA	NA	NA	NA	NA
*PbrbHLH24*	*PbrbHLH28*	WGD	WGD	NG	0.06	0.16	0.39	53.45	1.34E-08
*PbrbHLH25*	*PbrbHLH145*	WGD	WGD	NG	NA	0.01	0.00	2.39	NA
*PbrbHLH29*	*PbrbHLH72*	WGD	WGD	NG	0.59	2.37	0.25	790.30	9.99E-18
*PbrbHLH29*	*PbrbHLH88*	WGD	WGD	NG	0.06	0.22	0.28	73.35	5.03E-14
*PbrbHLH30*	*PbrbHLH138*	WGD	WGD	NG	0.07	0.16	0.46	53.44	0.0025151
*PbrbHLH38*	*PbrbHLH112*	WGD	proximal	NG	0.08	0.25	0.31	82.40	6.76E-11
*PbrbHLH40*	*PbrbHLH15*	WGD	WGD	NG	0.25	1.73	0.15	577.13	1.60E-33
*PbrbHLH40*	*PbrbHLH111*	WGD	WGD	NG	0.03	0.20	0.13	67.82	1.39E-13
*PbrbHLH42*	*PbrbHLH48*	WGD	WGD	NG	0.09	0.24	0.35	80.89	4.09E-06
*PbrbHLH44*	*PbrbHLH46*	WGD	WGD	NG	0.03	0.16	0.18	53.81	2.18E-15
*PbrbHLH49*	*PbrbHLH114*	WGD	WGD	NG	0.07	0.16	0.41	54.11	2.60E-08
*PbrbHLH53*	*PbrbHLH186*	WGD	WGD	NG	0.01	0.01	0.59	3.39	0.416491
*PbrbHLH61*	*PbrbHLH47*	WGD	WGD	NG	0.09	0.17	0.50	57.55	0.000638
*PbrbHLH61*	*PbrbHLH142*	WGD	WGD	NG	0.09	0.17	0.52	57.55	0.0010697
*PbrbHLH68*	*PbrbHLH130*	WGD	WGD	NG	0.03	0.11	0.30	37.23	3.36E-05
*PbrbHLH69*	*PbrbHLH190*	WGD	WGD	NG	0.07	0.15	0.47	49.46	3.18E-05
*PbrbHLH70*	*PbrbHLH4*	WGD	WGD	NG	0.06	0.20	0.30	66.52	2.02E-06
*PbrbHLH71*	*PbrbHLH63*	WGD	WGD	NG	0.01	0.04	0.38	13.25	0.0088433
*PbrbHLH72*	*PbrbHLH94*	WGD	WGD	NG	0.20	0.40	0.50	131.94	8.95E-06
*PbrbHLH73*	*PbrbHLH76*	WGD	proximal	NG	NA	NA	NA	NA	NA
*PbrbHLH74*	*PbrbHLH75*	proximal	WGD	NG	NA	NA	NA	NA	NA
*PbrbHLH74*	*PbrbHLH179*	proximal	WGD	NG	0.05	0.32	0.17	105.65	2.57E-15
*PbrbHLH75*	*PbrbHLH179*	WGD	WGD	NG	0.05	0.32	0.17	105.65	2.57E-15
*PbrbHLH81*	*PbrbHLH47*	WGD	WGD	NG	0.09	0.18	0.49	60.41	0.000206
*PbrbHLH81*	*PbrbHLH61*	WGD	WGD	NG	0.00	0.01	0.24	4.66	0.0623594
*PbrbHLH81*	*PbrbHLH142*	WGD	WGD	NG	0.09	0.18	0.50	60.41	0.0003516
*PbrbHLH83*	*PbrbHLH151*	WGD	WGD	NG	0.33	2.81	0.12	938.19	4.15E-23
*PbrbHLH86*	*PbrbHLH93*	WGD	WGD	NG	0.05	0.11	0.50	35.10	0.007392
*PbrbHLH90*	*PbrbHLH123*	WGD	WGD	NG	0.03	0.13	0.21	43.84	7.79E-10
*PbrbHLH91*	*PbrbHLH194*	WGD	WGD	NG	0.19	0.37	0.51	123.02	0.0001704
*PbrbHLH97*	*PbrbHLH98*	tandem	tandem	NG	NA	NA	NA	NA	NA
*PbrbHLH100*	*PbrbHLH5*	WGD	WGD	NG	0.05	0.17	0.28	56.22	1.41E-08
*PbrbHLH104*	*PbrbHLH45*	WGD	WGD	NG	0.07	0.10	0.70	32.29	0.185088
*PbrbHLH104*	*PbrbHLH122*	WGD	WGD	NG	0.00	NA	NA	NA	NA
*PbrbHLH110*	*PbrbHLH152*	WGD	WGD	NG	0.01	0.01	1.78	2.63	0.722276
*PbrbHLH115*	*PbrbHLH24*	WGD	WGD	NG	NA	0.00	0.00	1.38	NA
*PbrbHLH115*	*PbrbHLH28*	WGD	WGD	NG	0.06	0.16	0.39	53.45	1.34E-08
*PbrbHLH121*	*PbrbHLH46*	WGD	WGD	NG	0.03	0.16	0.18	53.81	2.18E-15
*PbrbHLH122*	*PbrbHLH45*	WGD	WGD	NG	0.07	0.10	0.69	32.39	0.140337
*PbrbHLH129*	*PbrbHLH58*	WGD	WGD	NG	0.03	0.22	0.11	74.60	6.27E-17
*PbrbHLH132*	*PbrbHLH82*	WGD	WGD	NG	0.10	0.23	0.43	75.83	0.0033683
*PbrbHLH133*	*PbrbHLH37*	WGD	WGD	NG	0.07	0.17	0.39	57.16	2.79E-06
*PbrbHLH134*	*PbrbHLH59*	WGD	WGD	NG	0.08	0.19	0.45	61.87	0.0024985
*PbrbHLH134*	*PbrbHLH170*	WGD	WGD	NG	0.44	1.98	0.22	661.66	2.55E-15
*PbrbHLH136*	*PbrbHLH29*	WGD	WGD	NG	0.36	1.49	0.24	497.24	6.84E-23
*PbrbHLH136*	*PbrbHLH72*	WGD	WGD	NG	0.52	1.81	0.29	603.96	1.79E-15
*PbrbHLH142*	*PbrbHLH47*	WGD	WGD	NG	0.00	0.01	0.16	4.40	0.0337635
*PbrbHLH143*	*PbrbHLH131*	WGD	WGD	NG	0.07	0.14	0.49	47.75	0.0087228
*PbrbHLH146*	*PbrbHLH31*	WGD	WGD	NG	0.04	0.22	0.18	73.41	5.22E-17
*PbrbHLH151*	*PbrbHLH109*	WGD	WGD	NG	0.05	0.15	0.32	48.85	0.0001836
*PbrbHLH151*	*PbrbHLH174*	WGD	WGD	NG	0.00	0.02	0.07	8.22	0.0100508
*PbrbHLH153*	*PbrbHLH54*	WGD	WGD	NG	0.16	0.21	0.73	71.01	0.0116334
*PbrbHLH155*	*PbrbHLH150*	WGD	WGD	NG	0.24	0.75	0.32	249.03	9.36E-21
*PbrbHLH157*	*PbrbHLH149*	WGD	WGD	NG	0.06	0.17	0.37	55.19	5.61E-10
*PbrbHLH170*	*PbrbHLH89*	WGD	WGD	NG	0.06	0.20	0.30	66.11	4.02E-06
*PbrbHLH171*	*PbrbHLH53*	WGD	WGD	NG	0.32	1.62	0.20	541.67	9.61E-24
*PbrbHLH174*	*PbrbHLH109*	WGD	WGD	NG	0.04	0.13	0.34	43.89	0.0005669
*PbrbHLH177*	*PbrbHLH26*	WGD	WGD	NG	0.34	2.62	0.13	874.92	4.32E-31
*PbrbHLH178*	*PbrbHLH147*	WGD	WGD	NG	0.01	0.00	1.40	1.42	0.772501
*PbrbHLH196*	*PbrbHLH168*	WGD	WGD	NG	0.06	0.10	0.59	31.88	0.0536712
*PbrbHLH197*	*PbrbHLH169*	WGD	WGD	NG	0.01	0.02	0.37	7.33	0.0941955

### Conserved motif analysis of *PbrbHLH* gene family

The types and composition of inner motifs mainly determine the protein function, and the evolutionary relationships among these PbrbHLH proteins were also determined by analyzing their conserved motifs. To further identify motif constructions of the PbrbHLH proteins, the online MEME program was used in this study to detect motif patterns. As showed in Fig. 2b, 20 conserved motifs with low E-values were recognized. The details of motif patterns were shown in Table S3. These composition patterns were nearly consistent with the phylogenetic analysis results, which were similar within the same group, but varying greatly between groups. Among PbrbHLHs, although pattern [#1,2] was detected in all members as the conserved motif pattern for bHLH TF in Chinese white pear, some of the other motifs were present only in certain groups, including motif #8 in group B, I and K; motif #10 in group F and O; motif #11 in group N; motif #9 in group S and motif #19 in T. However, some unique motifs patterns also only could be detected in specific subfamilies. Such as the pattern [#13,12,10,1,2,6,3,14] in clade F, the pattern [#15,7,5,18,1,2,6,3] in clade K and the pattern [#1,2,6,3,20] in clade J. We found that many subfamilies had relatively certain motif composition and there were significant differences among each other. However, there were some groups that have more than one pattern, and no conserved pattern was detected in some other clades, indicating that *PbrbHLHs* in these groups were not conservative in the evolutionary process, and the division among groups might have occurred in an early period.

### Expression profile and patterns of *PbrbHLH* genes in response to drought and cold stresses

**Fig. 4:**
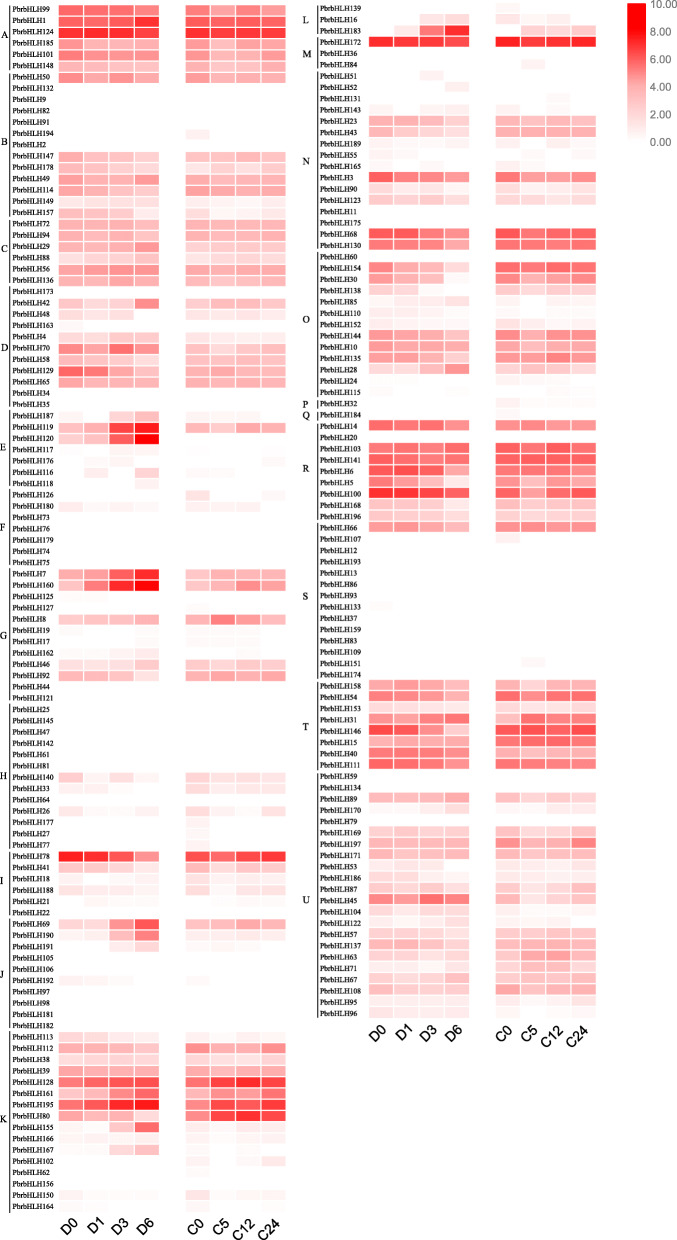
Expression profile of *PbrbHLHs* under drought and cold stresses. Expression analyses of *PbrbHLHs* using previous published transcriptome data under cold and drought stress conditions

Previous transcriptome data revealed the expression patterns of candidate *PbrbHLH* genes in response to drought stress and cold stress, respectively (Fig. 4) [35, 36]. Overall, the results indicated that although the background expression of some *PbrbHLH* genes was rarely detected, that of others was significantly different among these investigated time points. Several differentially expressed genes showed up-regulation trend under both two stress conditions, to varying degrees, such as *PbrbHLH119* and *PbrbHLH120* in clade E, *PbrbHLH7* and *PbrbHLH160* in clade G and *PbrbHLH128* to *PbrbHLH80* from clade K. This suggested that these genes may be involved in some close-related pathways in response to drought and cold stresses. Interestingly, compared to the expression of these genes in cold treatment, the peak expression of them under drought condition was showed at a relatively late time point. In contrast, some other *PbrbHLHs* showed different (or even opposite) expression patterns, indicating that their responses might vary according to the different stress conditions.

**Fig. 5:**
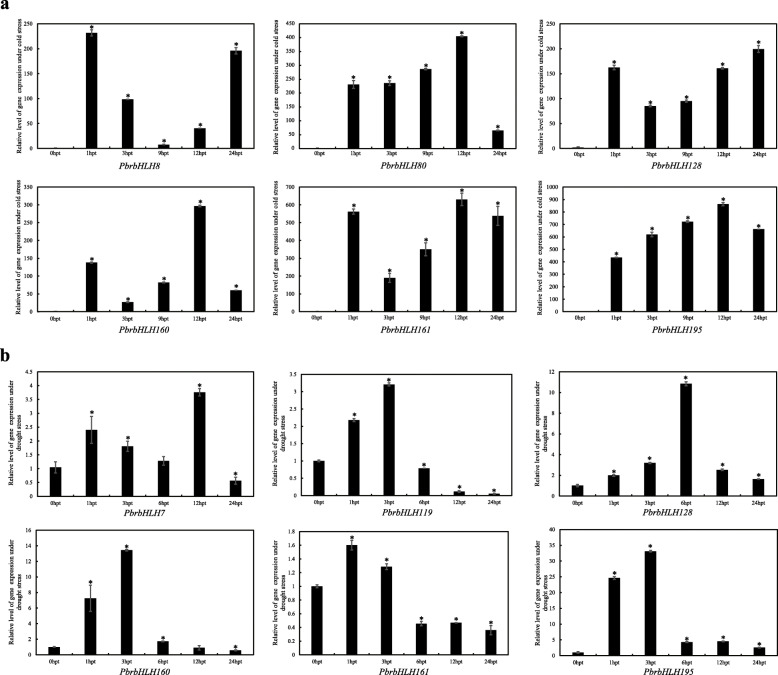
Expression analysis of *PbrbHLHs* undercold and drought stresses. **a** Relative expression of *PbrbHLH8*, *PbrbHLH80*, *PbrbHLH128*, *PbrbHLH160*, *PbrbHLH161* and *PbrbHLH195* with cold treatment. **b** Relative expression of *PbrbHLH7*, *PbrbHLH119*, *PbrbHLH128*, *PbrbHLH160*, *PbrbHLH161* and *PbrbHLH195* with drought treatment. The pear *Actin* was used as internal reference for the normalization. The statistical analyses were performed using student’s t-test (* *p* < 0.05)

To further verify the functions of these identified *PbrbHLHs*, eight differentially expressed *PbrbHLH* genes (*PbrbHLH119* from clade E, *PbrbHLH7*,*PbrbHLH8* and *PbrbHLH160* from clade G, *PbrbHLH80*, *PbrbHLH128*, *PbrbHLH161* and *PbrbHLH195* from clade K) were selected to examine the expression in response to drought and cold stresses, respectively (Fig. 5). Comparing with the expression at 0 hpt (hours post treatment), except *PbrbHLH8* and *PbrbHLH80* in drought treatment as well *PbrbHLH7* and *PbrbHLH119* under cold stress (data not shown), expressions for all other genes were significantly altered in the early stage of drought or cold treatment. Their responses tended to be more rapid under drought conditions, usually changing within the first 12 h. Under cold stress, expression of *PbrbHLH8*, *PbrbHLH128*, *PbrbHLH160* and *PbrbHLH161* initially showed down-regulating trend before being up-regulated as well as the expression of *PbrbHLH7* and *PbrbHLH195* under drought stress. The opposite trends between cold and drought stresses were noted for *PbrbHLH128* and *PbrbHLH160*. Under drought stress, both were up-regulated at first and then down-regulated, whereas, under cold stress, their expression initially decreased before increasing. These results indicated that *PbrbHLH* genes were indeed involved in the responses to drought and cold stresses, and the pathways they taken part in under these stresses condition seemed to be different.

### Silencing of *PbrbHLH195* reduced cold tolerance of *P. betulaefolia*

**Fig. 6:**
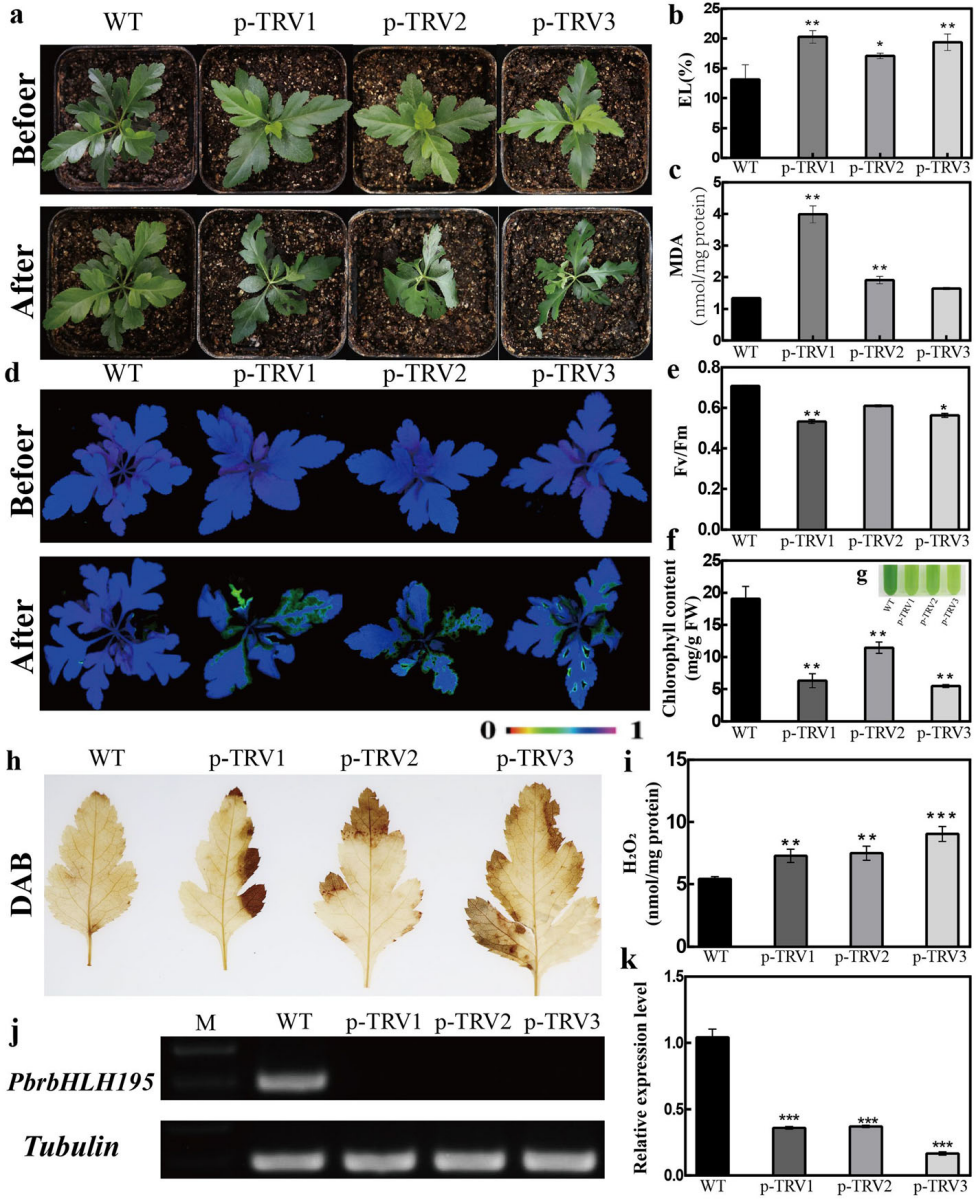
Cold tolerance assay of *PbrbHLH195*-silenced *Pyrus betulaefolia* plants. **a**-**c** Phenotype of 1-month-old *PbrbHLH195*-silenced plants before and after cold treatment for 8 days (**a**). Electrolyte leakage (EL) (**b**) and malondialdehyde (MDA) concentrations (**c**) after cold treatment. **d**-**g** Chlorophyll fluorescence imaging of silenced plants and WT plants(**d**), Fv/Fm ratios (**e**), Chl content (**f**) and phenotype (**g**) of WT and *pTRV-PbrbHLH195* silencing plants (pTRV-1, pTRV-2 and pTRV-3) at the end of the chilling stress. **h**-**i** In situ accumulation of H_2_O_2_ of WT and silencing plants, as revealed by histochemical staining with 3, 3-diaminobenzidine (DAB) (**h**) after cold treatment. Quantitative measurement of H_2_O_2_ (**i**) levels after cold treatment. The expression of *PbrbHLH195* was detected by RT-PCR (**j**) and qRT-PCR (**k**) at 8d after cold treatment

To understand whether *PbrbHLHs* is required for cold tolerance in pear, the VIGS system was employed to silence *PbrbHLH195*, which is significantly up-regulated under cold condition, in *P. betulaefolia*. The transcript abundance of *PbrbHLH195* in the positive plants was substantially reduced by 50–90%, compared with that of WT (Fig. 6j, k). The positive silent plants (p-TRV1, p-TRV2 and p-TRV3) and WT plants were morphologically indistinguishable under normal growing conditions (Fig. 6a, d). However, upon exposure to 0 °C for 8 days, the silent plants displayed more severe damage in comparison with WT (Fig. 6a). The electrolyte leakage (EL) and malondialdehyde (MDA) concentrations in silent plants were significantly higher than those in WT under cold stress (Fig. 6b, c). Meanwhile, when they were subjected to cold treatment, Chl fluorescence in silent plants were prominently repressed, accompanied by significantly lower Fv/Fm ratio and Chl content, in comparison with WT (Fig. 6d-g). In addition, compared with silent plants, WT had lower H_2_O_2_ content (Fig. 6h, i). In situ accumulation of H_2_O_2_ was histochemically stained with DAB. In the presence of low temperature, the staining became darker, but silent plants staining was deeper and stronger than that of WT (Fig. 6h, i), which was further confirmed by quantitative measurement (Fig. 6i), which means that silencing plants accumulate more reactive oxygen species than WT. These results indicated that silencing of *PbrbHLH195* promotes cold susceptibility in *P. betulaefolia*.

## Discussion

After the release of the Chinese white pear genome sequencing data, there were many TF genes at the whole-genome level have been identified and characterized, including NAC-TF, BAM-TF and WRKY-TF et.al [22, 37, 38].. bHLH transcription factors are involved in many pathways in plant growth and metabolism [12]. However, no such detailed studies have been done with the *bHLH* family, and only a few examinations have been made of *PbrbHLHs* in pear. Here, we identified 197 *PbrbHLH* genes in Chinese white pear. Results of the phylogenetic analysis, gene structure and protein conserved motif analysis enable us to classify these PbrbHLH proteins into 21 groups, which is the same number reported in tomato and apple [28, 39], even though those organisms have fewer *SlbHLHs* (159) and *MdbHLHs* (188) than the members of *PbrbHLHs* in pear. On the basis of phylogenetic analysis, the un-rooted tree showed that *PbrbHLHs* were well separated into 19 clades with the wildly varied gene numbers from 3 (clade L and M) to 22 (clade U) and two one-gene clade P and Q. The gene and protein structure analysis showed that *PbrbHLH* family also has a broad diversity in intron/exon organizations as well the protein motif patterns. Although, the distribution pattern of exons and UTRs in clade D, F, G, H, J, K and U were relatively conserved, there was a broad range of exon numbers and structural diversity in many other clades, which is similar to the results of protein motif pattern analysis. By using online MEME software, 20 conserved protein motifs were detected among PbrbHLHs with low E values, and pattern [#1,2] were existed in all bHLHs which was regarded as the characteristic pattern for PbrbHLH TF. Meanwhile, some other motifs were present only in certain groups, including the motif #8 in group B, I and K and motif #10 in group F and O. Furthermore, three unique motif patterns only could be detected in specific subfamilies, respectively, such as the pattern [#13,12,10,1,2,6,3,14] in clade F, the pattern [#15,7,5,18,1,2,6,3] in clade K and the pattern [#1,2,6,3,20] in clade J. These results suggested that the *PbrbHLH* gene family may play diverse roles in the adaptive evolution to environmental stresses, and the division among groups might have occurred in an early period.

Gene duplication analysis revealed that the main driving force for the expansion of *PbrbHLH* family was WGD/segmental events, which is same as the case in apple. For instance, by applying MCScanX, 58.9% of *bHLH* genes in Chinese white pear were categorized into WGD/segmental type. Although pear was undergone the recent WGD events, almost one quarter of *bHLH* genes were duplicated from dispersed events. This may be due to the high ratio of self-incompatibility and the domestication process of pear. These results showed that WGD/segmental and dispersed gene duplications play critical roles in the expansion of the *bHLH* gene family in Chinese white pear. Furthermore, Ks values analysis implied that almost all WGD type *PbrbHLH* genes were duplicated from a date around the recent WGD event, and the Ka/Ks ratios indicated that *PbrbHLHs* evolved mainly under purifying selection and they seem to be necessary for adaptation to the current environment in their evolutionary history.

The function enrichment analysis showed that *PbrbHLH* genes were mainly enriched in the functions and processes closely related to TF, and the pathways they classified in were the main mechanisms by which *bHLH* family TFs regulate the expression of downstream genes, such as circadian rhythm, MAPK signaling and plant hormone signal transduction pathways. For example, *OsbHLH148* and *OsbHLH006* (REJ1) can improve drought stress by jasmonic acid signaling pathway in rice. Under salt and drought stress, in grapes *VvbHLH1* confers a dominant effect on salinity and drought tolerance thought increasing the accumulation of flavonoids and ABA signaling in transgenic *Arabidopsis thaliana*. In addition, bHLH protein is also involved in plant stress resistance. *Arabidopsis AtbHLH112* gene improves drought tolerance by increasing osmotic substances, eliminating ROS content and reducing water diversion. The results indicated that *PbrbHLHs* might play roles as other *bHLHs*.

By analyzing previous transcriptome data, we revealed the expression patterns of *PbrbHLHs* under cold and drought stress conditions. The results showed that, except some genes, the expression of most *PbrbHLHs* was significantly altered. For example, under both two stresses, *PbrbHLH* genes including *PbrbHLH7*, *PbrbHLH119*, *PbrbHLH120*, *PbrbHLH160* and *PbrbHLH128* to *PbrbHLH80* in clade K had an up-regulation trend, which suggested that these genes might play similar roles in some close-related pathways in response to drought and cold stresses. Comparing with cold treatment, the peak expression of them under drought condition was showed at a relatively late time point, indicating that the responses of *PbrbHLHs* varied according to the treatment applied. To verify whether *PbrbHLHs* were involved in the response to cold or drought stresses, we performed stress treatments and qRT-PCR analysis. The results showed that the expression of all tested genes was significantly altered in the early stage of drought or cold treatments, however, the responses of same gene between two treatmentes could be diverse. For instance, under cold treatment, expression of *PbrbHLH7*, *PbrbHLH8*, *PbrbHLH161*, *PbrbHLH128*, *PbrbHLH160* and *PbrbHLH195* showed down-regulating trend at first before being up-regulated, whereas, under drought stress, *PbrbHLH128* and *PbrbHLH160* were up-regulated at first and then down-regulated. Furthermore, as a high up-regulated gene induced in both cold and drought stress conditions, the interference of *PbrbHLH195* in transcription level significantly reduced the cold tolerance of the RNAi pear seedlings. These results indicate that *PbrbHLH* genes were involved in the responses to drought and cold stresses in pear, and the pathways they involved in seemed to be different under various stress conditions.

Our works in this study highlight the importance of bHLH TF in the cold and drought tolerance of pear. This is the first study to identify the *PbrbHLH* genes and examine their expression patterns in pear. QRT-PCR analysis showed that *PbrbHLH* is involved in stress tolerance pathways and functional analysis showed that *PbrbHLH195* plays an important role in pear abiotic stress tolerance. However, further investigation will be required to understand the roles of *PbrbHLHs* in the stress response pathways, and the characterization of key (even the marker) bHLH TFs under each stress condition was also crucial to the revealing of the functional mechanisms of bHLH in pear.

## Conclusions

In this study, we identified 197 *PbrbHLH* genes from Chinese white pear and carried out phylogenetic analysis to determine the relationships among these genes. Based on the results of protein motifs and intron/exon characteristics and phylogenetic analysis, *PbrbHLH* family was classified into 21 groups. According to the analysis of collinearity, WGD and dispersed duplication might have a role in the evolution of the *PbrbHLH* family. In addition, RNA-seq data, qRT-PCR and VIGS results revealed that *PbrbHLH* genes might have important roles in response to abiotic stresses, and the expression patterns of them differed in response to drought and cold stresses. The underlined collected data from this study provided a foundation for advanced studies to evaluate the mechanisms of cold-tolerance and drought-tolerance for *bHLH* genes in pear.

## Methods

### Plant materials and bacterial strains

The pear seedlings were grown in the greenhouse under a 16 h/8 h light/dark photoperiod, 75% relative humidity and 25 °C. *A. tumefaciens* GV3101 was grown in LB media supplemented with kanamycin and Rif at 28 °C in an orbital shaker at 200 rpm and harvested during the log phase of growth for infiltration.

### Identification and functional annotation of *bHLH* gene family in Chinese white pear

To identify the *bHLH* genes in Chinese white pear, we performed multiple database-based searches. We downloaded all needed sequences and annotation file of Chinese white pear from Pear Centre of Nanjing Agricultural University (http://peargenome.njau.edu.cn/) and the seed file of bHLH conserved domain (PF00010) was downloaded from Pfam (http://pfam.sanger.ac.uk/). HMMER (Hidden Markov Model, HMM) software was used to detect conserved Pfam domain with default parameters E-value < 0.05 [40]. Then we checked the predicted bHLH transcription factors by using the NCBI Batch CD-Search tools (Batch CD-Search: https://www.ncbi.nlm.nih.gov/Structure/bwrpsb/bwrpsb.cgi) based on CDD v3.18 and SMART v6.0 databases to verify the existence of bHLH domain (Table S1). The proteins with E-values greater than 1e^− 6^ or without a bHLH domain were removed. The relevant gene ID of *PbrbHLH* genes were shown in Table 1. The annotation information for Chinese white pear was extracted from the GFF file, and the result was visualized by a R script. The BLASTP was performed against 167 reported AtbHLH protein sequences [5], and the protein sequences were downloaded from TAIR (The Arabidopsis Information Resource, https://www.arabidopsis.org/).

### Structure and conserved motif analysis of the *PbrbHLH* genes

The Gene Structure Display Server (GSDS 2.0) (http://gsds.cbi.pku.edu.cn/) was used to analyze the structures of the *bHLH* genes by aligning the cDNA sequences with their corresponding genomic DNA sequences [41]. Conserved motif analysis of bHLH proteins was performed by online Multiple Expectation Maximization for Motif Elicitation (MEME) (http://meme.nbcr.net/meme/cgibin/meme.cgi) with default parameters, and maximum number parameter of motifs were set as 20 [42]..

### Phylogenetic analysis

The phylogenetic tree was built with Neighbor-Joining (NJ) method and a bootstrap of 1000 in MEGA7.0 (http://www.megasoftware.net/) [43]. The p-distance was used and the optional parameters for pairwise deletion were considered.

### Chromosomal localization and synteny analysis

The chromosomal localization information was extracted from the GFF file. The same procedure used in the PGDD (http://chibba.agtec.uga.edu/duplication/) was performed to analyze the synteny among the *PbrbHLHs*. Primarily, the local all-vs-all BLASTP searches among identified *PbrbHLH* genes were conducted (E < 1e^− 10^). Afterward, MCScanX was employed for the determination of syntenic gene pairs with the BLASTP result and gene location information used as input files [44]. The downstream analysis tool (duplicate_gene_classifier) in the MCScanX package was employed for the identification of tandem, proximal dispersed, and segmental/whole-genome duplications (WGD) of *PbrbHLH* family genes. The results were visualized using circos-0.69 software [45]. The Ka and Ks values were analyzed via KaKs_Calculator 2.0 [46]. For the estimation of the date of segmental duplication events, the succeeding pairs of homologous genes within 100 Kb on all sides of the *PbrbHLH* genes, considered for the mean Ks calculation.

### Expression analysis of *PbrbHLH* genes under drought and cold stress conditions

Published transcriptomic data (FPKM values) characterizing the total RNA of drought treatment samples (D0, D1, D3 and D6 indicating the samples harvested at 0 hpt (hour post treatment), 1 hpt, 3 hpt and 6 hpt under drought stress) were downloaded from Li et al. (2016) [35]; cold treatment samples (C0, C5, C12 and C24 indicating the samples harvested at 0 hpt, 5 hpt, 12 hpt and 24 hpt under cold stress) were downloaded from Yang and Huang (2018) [36]. We determined the expression patterns of *PbrbHLH* family genes under drought and cold stress conditions. The differentially expressed genes were identified with the threshold |log2^FC^| > 1. TBtools v1.068 was used to visualize the results [47].

For the qRT-PCR analysis, 9-week-old pear seedlings were treated with drought and cold, respectively. The leaves were cryopreserved with liquid nitrogen at 0 hpt, 1 hpt, 3 hpt, 6 hpt, 12 hpt and 24 hpt after drought stress treatment as well the leaves with cold treatment at 0 hpt, 1 hpt, 3 hpt,9 hpt, 12 hpt and 24 hpt. Total RNA extraction and the synthesis of cDNA were according to the instructions of RNA kit (Tiangen, Beijing, China) and PrimeScript RT reagent Kit (Trans Gen). Specialized primers of the constitutive *TUB* and eight tested *PbrbHLH* genes were designed via NCBI online tool Primer-BLAST (https://www.ncbi.nlm.nih.gov/tools/primer-blast/index.cgi?LINK_LOC=BlastHome) with the Specificity Parameters Organism option set as *Pyrus bretschneideri* (taxid:225117) (Table S4). The qRT-PCR assays were conducted with three technical copies. QRT-PCR reactions (20 μl per hole) were performed as previously reported [48]. The expression was evaluated for each sample via the 2^−ΔΔCt^ method, and Duncan’s multiple range test was conducted. A *p*-value of less than 0.05 was the considerable variation and the differentially expressed genes were identified with |log2^FC^| > 1.

### Generation of silenced plants

Virus-induced gene silencing (VIGS)-mediated suppression of *PbrbHLH195* was performed according to previous methods [49]. A 182 bp fragment of *PbrbHLH195* open reading frame (ORF) was inserted into *EcoR* I and *BamH* I sites of tobacco rattle virus-based vector 2 (TRV2) to generate the pTRV2-*PbrbHLH195* construct. The vectors pTRV1, pTRV2 and pTRV2-*PbrbHLH195* were transformed into *A. tumefaciens* strain GV3101 by heat shock. The bacterial cells (OD600 = 1.0) containing pTRV1 were mixed with pTRV2-*PbrbHLH195* or pTRV2 in a 1: 1 volume ratio in 2-(Nmorpholino) ethanesulfonic acid (MES) buffer (10 mM MgCl_2_, 200 mM acetosyringone, and 10 mM MES, pH 5.6) and kept slowly shaking in dark for 4 h at room temperature. The bacterial mixtures were injected into the leaves of seedlings and rinsed with water, grown in soil pots and transferred to a controlled growth chamber. Two weeks later, un-injected leaves were collected from each plant and subjected to genomic PCR and qRT-PCR analyses, and the VIGS plants exhibiting similar magnitude of *PbrbHLH195* suppression were used for further analyses.

### Physiological analysis

EL was measured according to [50]. MDA and H_2_O_2_ were detected according to the instructions using the corresponding detection kit (Nanjing Jiancheng Bioengineering Institute, Nanjing, China). Chl fluorescence was measured by Imaging PAM CHL fluorometer, Fv/FM ratio was calculated by imaging Winge software (Walz, Germany). Chl was extracted and analyzed according to [51].

## Supplementary Information

**Additional file 1 Table S1.** Detailed characteristics of *PbrbHLHs*.**Table S2.** Duplication type of *PbrbHLH* genes in Chinese white pear. **Table S3.** Sequence informations of 20 detected motifs in MEME analysis. **Table S4.** The primers of *PbrbHLHs* for qRT-PCR and vector construction.

**Additional file 2 Fig. S1.** Phylogenetic tree of 167 AtbHLHs and the two unique PbrbHLH proteins. MEGA 7 was used to construct the phylogenetic tree based on the protein sequences. iTOL was used to annotate and review the phylogenic tree. **Fig. S2.** Functional annotation enrichment analysis. (a) GO (Gene ontology) term enrichment analysis of PbrbHLH proteins. (b) KEGG enrichment analysis of PbrbHLH proteins.

## Data Availability

All needed genome sequences and genome annotation files of Chinese white pear were obtained from the Nanjing Agricultural University pear genome project website (http://peargenome.njau.edu.cn). All data generated or analysed during this study are included in this published article and its supplementary information files.

## Abbreviations

bHLH: Basic helix-loop-helix
TF: Transcription factor
*COR* gene: Cold regulated gene
pI: Protein isoelectric points
GRAVY: Grand average of hydropathy
Ks: Synonymous substitutions per site
hpt: Hours post treatment
EL: Electrolyte leakage
MDA: Malondialdehyde
HMM: Hidden Markov Model
GSDS: Gene Structure Display Server
MEME: Multiple Expectation Maximization for Motif Elicitation
NJ: Neighbor-Joining
RPKM: Fragments per kilobase of exon model per million mapped reads
WGD: Whole-genome duplications
VIGS: Virus-induced gene silencing
ORF: Open reading frame
MES: 2-(Nmorpholino) ethanesulfonic acid
