# Supplementary material for: Genome-wide identification of PbrbHLH family genes, and expression analysis in response to drought and cold stresses in pear (Pyrus bretschneideri)
Source: BMC Plant Biol. 2021 Feb 9;21:86. doi: 10.1186/s12870-021-02862-5 (PMC7874673; doi:10.1186/s12870-021-02862-5)

Genome-wide identification of *PbrbHLH* family genes, and expression analysis in response to drought and cold stresses in pear (*Pyrus bretschneideri*)

Huizhen Dong[1,2], Qiming Chen[1,2], Yuqin Dai, Wenjie Hu, Shaoling Zhang* and Xiaosan Huang*

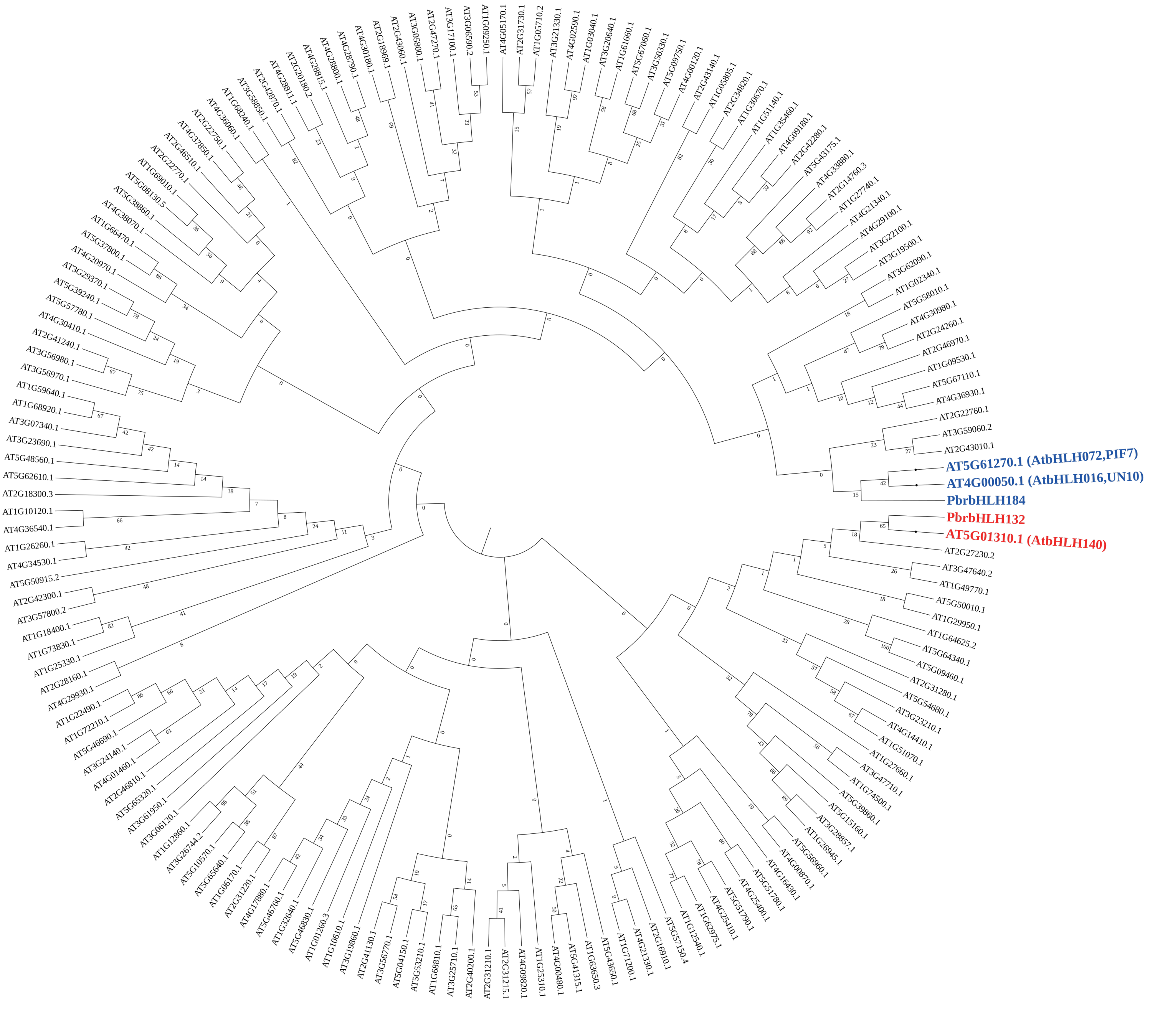

Genome-wide identification of *PbrbHLH* family genes, and expression analysis in response to drought and cold stresses in pear (*Pyrus bretschneideri*)

Huizhen Dong[1,2], Qiming Chen[1,2], Yuqin Dai, Wenjie Hu, Shaoling Zhang* and Xiaosan Huang*

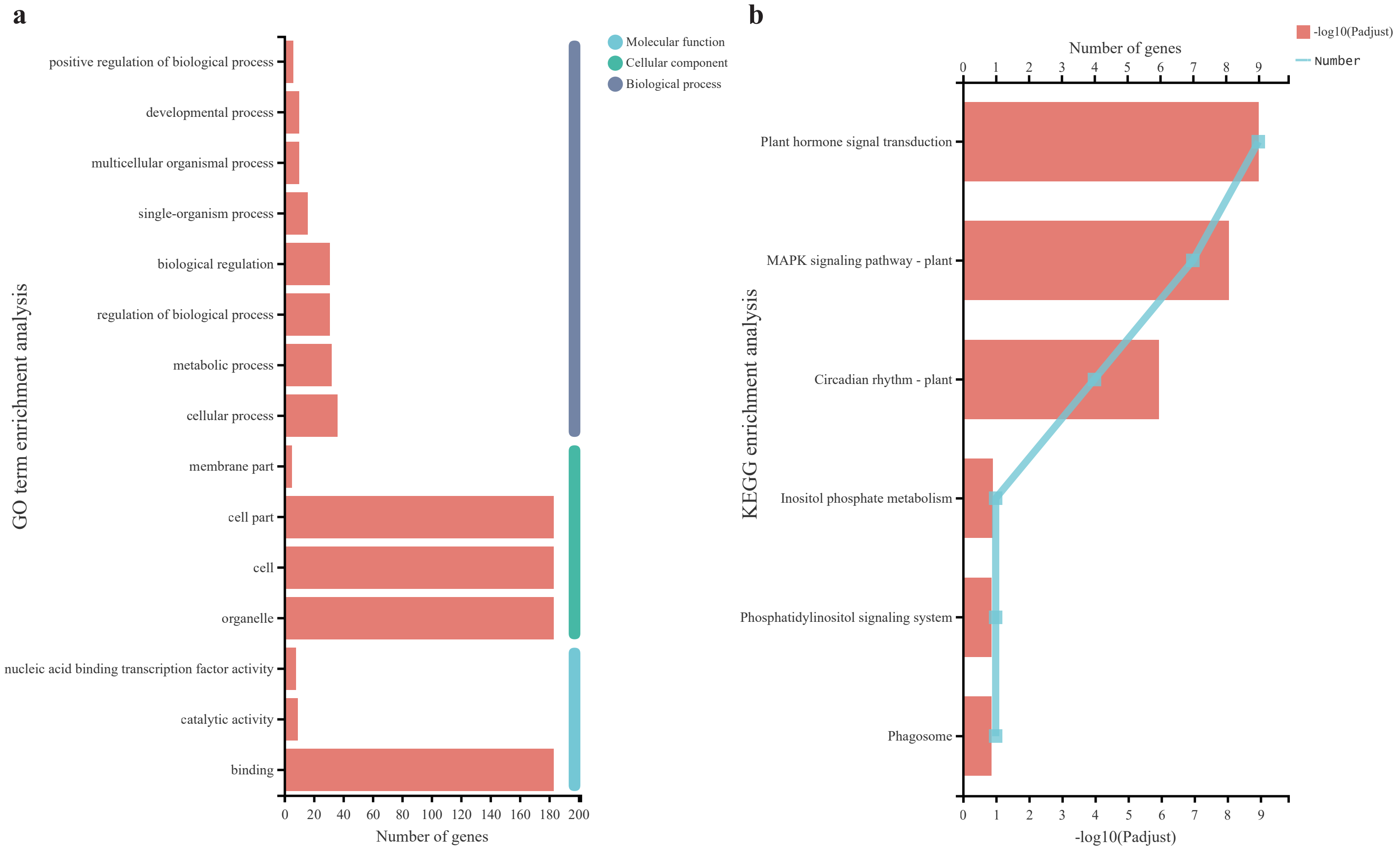

Supplement: Supplementary file 2 — Additional file 2 Fig. S1. Phylogenetic tree of 167 AtbHLHs and the two unique PbrbHLH proteins. MEGA 7 was used to construct the phylogenetic tree based on the protein sequences. iTOL was used to annotate and review the phylogenic tree. Fig. S2. Functional annotation enrichment analysis. (a) GO (Gene ontology) term enrichment analysis of PbrbHLH proteins. (b) KEGG enrichment analysis of PbrbHLH proteins. [file 12870_2021_2862_MOESM2_ESM.pdf]
